# Efficacy of Probiotic Strains *Lactobacillus sakei* Probio65 and *Lactobacillus plantarum* Probio-093 in Management of Obesity: An In Vitro and In Vivo Analysis

**DOI:** 10.3390/ph17060676

**Published:** 2024-05-24

**Authors:** Aneela Gulnaz, Lee-Ching Lew, Yong-Ha Park, Jamal S. M. Sabir, Raed Albiheyri, Irfan A. Rather, Yan-Yan Hor

**Affiliations:** 1 Department of Biotechnology, Yeungnam University, 280 Daehak-ro, Gyeongsan 38541, Gyeongbuk, Republic of Korea; 2 Probionic Corp., Jeonbuk Institute for Food-Bioindustry, 111-18, Wonjangdong-gil, Deokjin-gu, Jeonju-si 38541, Jeollabuk-do, Republic of Korea; 3 Department of Biological Sciences, Faculty of Science, King Abdulaziz University, Jeddah 21589, Saudi Arabia; 4 Centre of Excellence in Bionanoscience Research, King Abdulaziz University, Jeddah 21589, Saudi Arabia

**Keywords:** obesity, adipocyte, *Lactobacillus*, probiotics, triglycerides, prevention

## Abstract

The prevalence of obesity, characterized by an excessive accumulation of adipose tissue and adipocyte hypertrophy, presents a major public health challenge. This study investigates the therapeutic potential of two probiotic strains, *Lactobacillus sakei* Probio65 and *Lactobacillus plantarum* Probio-093, in the context of obesity. Utilizing 3T3-L1 cell-derived human adipocytes, we assessed Probio65’s and Probio-093’s capacity to mitigate triglyceride accumulation and influence adipocytokine production in vitro. Subsequently, an in vivo trial with male C57BL/6J mice examined the effects of both probiotic strains on adipose tissue characteristics, body weight, fat mass, and obesity-related gene expression. This study employed both live and ethanol-extracted bacterial cells. The results demonstrated significant reductions in the triglyceride deposition, body weight, and adipose tissue mass in the treated groups (*p* < 0.05). Furthermore, both strains modulated adipokine profiles by downregulating proinflammatory markers such as PAI-1, leptin, TNF-α, STAMP2, F4/80, resistin, and MCP-1, and upregulating the insulin-sensitive transporter GLUT4 and the anti-inflammatory adiponectin (*p* < 0.05). Our findings suggest that *Lactobacillus sakei* Probio65 and *Lactobacillus plantarum* Probio-093 are promising agents for microbiome-targeted anti-obesity therapies, offering the effective mitigation of obesity and improvement in adipocyte function in a murine model.

## 1. Introduction

Obesity is a chronic condition and a complex disease reflected by an excessiveness of body fat, expanded adiposity, and altered cell composition of adipose tissues. Additionally, it is interconnected alongside an inflated risk of various health conditions, including high blood pressure, cardiac arrest, asthma, diabetes, and several types of cancer [1,2,3,4]. Adipose tissue (AT) is a metabolic body part that plays a crucial role in controlling energy homeostasis. AT comprises lipid-rich cells called adipocytes, which are responsible for energy storage in the form of lipids, as well as regulating sugar metabolism and insulin sensitivity through the release of bioactive peptides called adipokines [5,6]. In obesity, however, the energy storage capacity of adipocytes increases, leading to the enlargement of fat tissue and the hypertrophy (overgrowth) of adipocytes. These enlarged adipocytes secrete adipokines that contribute to insulin resistance and show impaired energy expenditure [7,8,9,10]. This state of chronic energy excess leads to the expulsion of fatty acids into the circulation, which accumulate in the inside of other organs and disrupt metabolism. It appears that the adipocytes of obese individuals release free fatty acids even in the presence of insulin [11].

Numerous studies have demonstrated that obesity is often caused by the excessive consumption of high-fat diet (HFD) foods, leading to the expansion and alteration of fat cells, resulting in inflammation and oxidative stress [1,2,4,12]. Inflammation, closely associated with insulin resistance and metabolic dysregulation induced by obesity, can be effectively mitigated by selectively targeting and inhibiting key inflammatory pathways within AT [4,13,14,15,16,17,18]. Indications, for instance, body mass size, dietary factors, and excessive caloric intake, exert a significant influence on the cellular architecture of healthy AT, and especially on the configuration of its immune cells. These factors can contribute to metabolic disturbances, resulting in peripheral insulin resistance, systemic inflammation, and the intrusion of macrophages into adipose cells [2,4,13,14,15,19].

Probiotics, when properly consumed in moderate amounts, confer health benefits to the host, and have been shown to have potential in preventing metabolic syndromes such as obesity [20,21,22,23,24,25,26,27,28,29,30]. The most cited probiotic types are *Lactobacillus* and *Bifidobacterium* genera, with *Lactobacillus* having positive effects on the body mass index, glucose metabolism, inflammation, and host immunity [31]. Recent studies have also demonstrated that probiotics can convert compounded complex dietary polysaccharides into short-chain fatty acids (SCFAs), which are vital for strengthening metabolic disturbances and metabolic syndrome-associated disorders [32,33,34,35,36,37,38]. These SCFAs act at various levels to decrease inflammatory conditions, reduce insulin resistance, improve insulin discharge and glucagon-like peptide (GLP-1) excretion, and enhance β-cell function [39,40,41]. Modulating the composition of the intestinal microbiota through probiotic ingestion is thus a potential approach for treating obesity and lowering inflammation levels in AT. Probiotics have many applications and are commonly used as functional ingredients in medicine, in feed and food industries, and some more examples include starter cultures in dairy products and as antimicrobial agents and biopreservatives. There are also many health benefits of probiotics, mostly in the area of gastrointestinal health, such as reducing diarrhea and protecting against colon cancer. In recent years, the benefits of probiotics have been expanded beyond the gut and include improving skin inflammation, allergic diseases, mood, atherosclerosis, NAFLD, osteoporosis, and metabolism-related diseases, which are the focus of our project on the management of obesity [42].

The objective of this research was to explore the potential anti-obesity influences of two distinct probiotic strains, namely, *Lactobacillus sakei* Probio65 and *Lactobacillus plantarum* Probio-093, using in vivo and in vitro analysis of AT. As obesity remains a major concern for public health globally, affecting an increasing number of people every year, it is crucial to explore all possible solutions in the fight against this disease.

## 2. Results

### 2.1. Triglyceride Contents (TG)

**Figure 1:**
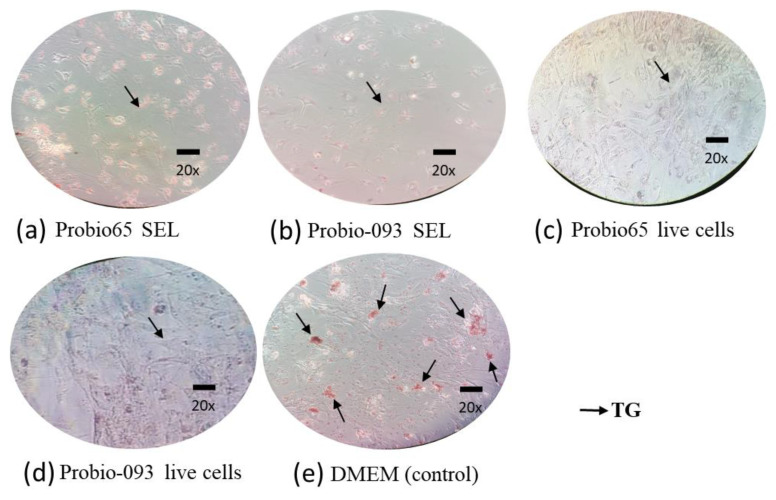
Microscope images (under magnification of 20×) of 3T3-L1 cells treated with Probio65 and Probio-093 on the last day of differentiation to see intracellular triglyceride accumulation in adipose tissue cell line. (**a**) Probio65 SEL: fully differentiated 3T3L1 cells treated with Probio65 SEL (100 µL/mL). (**b**) Probio-093 SEL: fully differentiated 3T3L1 cells treated with Probio-093 SEL (100 µL/mL). (**c**) Probio65 live cells: completely differentiated 3T3L1 cells treated with Probio65 (10^8^ CFU). (**d**) Probio-093 live cells: thoroughly differentialized 3T3L1 cells treated with Probio-093 (10^8^ CFU). (**e**) DMEM control group: entirely differentiated 3T3L1 cells treated with DMEM as a negative control.

We examined the deposition of lipid droplets in the 3T3-L1 cells and the red coloration is evidence of lipid accumulation in the cells. The microscopic evaluation showed a much lower presence of lipid droplets in the 3T3-L1 cells treated with both the live cells and SEL of Probio-093 and Probio65. In the untreated cells (3T3-L1) cultured with DMEM media only (control group), all the cells accumulated lipid droplets of different sizes. Such a result serves as a qualitative measure of the intracellular lipid contents (Figure 1).

### 2.2. Gene Expression—In Vitro

We also measured the mRNA expression levels of some metabolic key regulatory genes, such as markers for PAI, MCP-1, TNF-α, resistin, leptin, STAMP2, F4/80, GLUT4 (Glucose Transporter type 4), and adiponectin, in 3T3-L1-derived differentiated adipocytes. The total gene expression results were compared to their respective controls, normalized by β-actin, and expressed as relative trends. Adipose tissues before differentiation, like normal adipose cells, were used as a PC (positive control) and fully differentiated cells containing only DMEM media were considered a NC (negative control).

**Figure 2:**
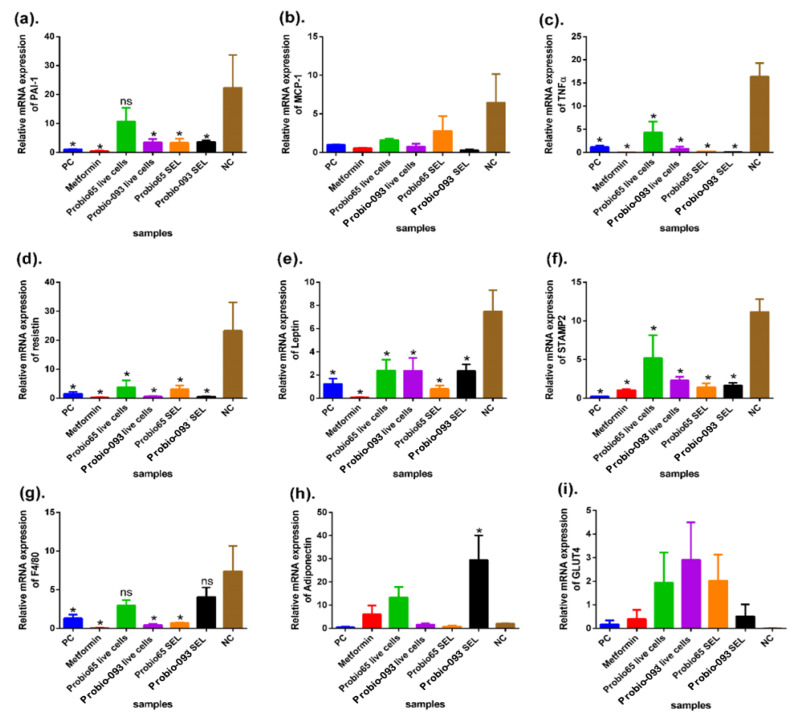
The relative expression of the genes (**a**) PAI-1, (**b**) MCP-1, (**c**) TNF-α, (**d**) resistin, (**e**) leptin, (**f**) STAMP2, (**g**) F4/80, (**h**) adiponectin, and (**i**) GLUT4 in 3T3-L1 cells was analyzed. Statistical distinction (* *p* < 0.05) was specified by contrasting the results with the negative control (NC) group. ns indicate no sigificance. All the gene expression results were compared to their respective controls, normalized by β-actin, and quantified as relative expression.

According to Figure 2a, 3T3L1 adipose tissues treated with live cells and the SEL of Probio-093 and SEL of Probio65 showed a significantly lower expression of PAI-1 (*p* < 0.05). However, no notable differentiation was seen in the cells treated with live cells of Probio65 as compared with the NC group of cells.

In reference to the data depicted in Figure 2b, it becomes apparent that there is a lack of substantial differentiation in MCP-1 expression across all the treatment cohorts, namely, PC, metformin, live cells of Probio65, SEL of Probio65, live cells of Probio-093, and SEL of Probio-093, in comparison with the NC group.

The relative gene expression of TNF-α in the 3T3L1 cell line is presented in Figure 2c. According to the data, the PC cells-, live cells and SEL of Probio-093-, and the SEL and live cells of Probio65-treated cells exhibited significantly decreased TNF-α expression relative to the NC group (*p* < 0.05).

Figure 2d represents the expression of resistin, as it was markedly lowered in 3T3L1 cells by the treatment of metformin, live cells, and the SEL of both the Probio-093 and Probio65 strains (*p* < 0.05) as compared to the NC group.

The relative gene expression of leptin in 3T3L1 cells is displayed in Figure 2e. The results revealed that the 3T3L1 cells treated with the SEL and live cells of Probio65 and Probio-093 and metformin displayed notably reduced the gene expression in comparison with the NC (*p* < 0.05).

Figure 2f indicates the proportional mRNA expression of STAMP2 in the 3T3L1 adipose tissues. The PC group and all the treatment groups, metformin, live cells, and SEL of Probio-093 and Probio65, showed a similar trend of a remarkable decrease in gene expression (*p* < 0.05) when compared to the NC group.

The relative expression of the mRNA levels of F4/80 in the 3T3L1 cells was tremendously downregulated in the PC-, Probio-093 live cell-, Probio65 SEL- and metformin-treated cells (*p* < 0.05) in relation to the NC group (Figure 2g). The cells administered with Probio-093 SEL and Probio65 live cells had no notable difference when compared with the NC group.

According to Figure 2h, the expression of adiponectin was significantly upregulated in the Probio-093 SEL group (*p* < 0.05) compared to the NC group. Meanwhile, the cells treated with Probio65 live cells and SEL, Probio-093 live cells, and metformin, and the PC group exhibited no significant difference compared to the NC cells.

The data in Figure 2i confirmed the relative mRNA expression of GLUT4. There were no significant changes observed in the normal adipose 3T3L1 cells (PC) and any treatment group in comparison with the NC group.

### 2.3. Transitions in Murine Body Weight

**Figure 3:**
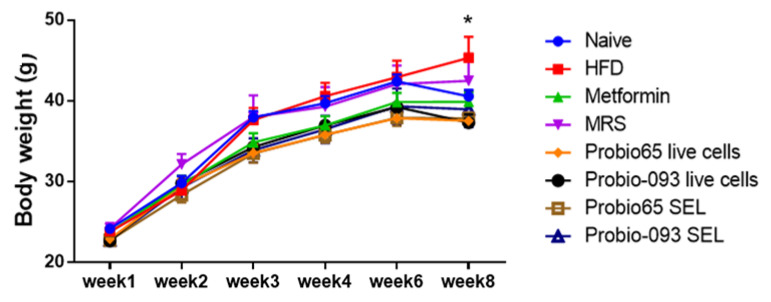
Transitions in murine body weight. Weekly fluctuations in body weight of mice administered with Probio65 and Probio-093 for 8 weeks, along with consumption of a high-fat diet (HFD). Statistical significance (* *p* < 0.05) specifies a significant differentiation compared with the HFD group.

Figure 3 depicts the change in body weight with time. Starting from baseline, HFD consistently elevated the body weight of mice on a weekly basis. We observed a significant difference between the treatment groups and the HFD group in week 8. The highest-weighing groups were the HFD and MRS-HFD group. In comparison, all the other treatment groups showed significantly lower body weights (* *p* < 0.05).

### 2.4. Adipose Weight Measurement

**Figure 4:**
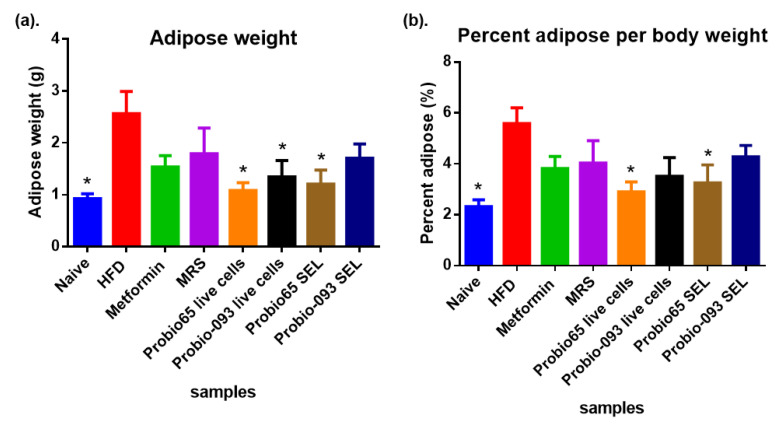
Assessment of adipose weight and percentage in mice treated with Probio65 and Probio-093 along with HFD. (**a**) Adipose weight measurement of mice administered Probio-093 and Probio65 for 8 weeks along with a high-fat diet (HFD). (**b**) Percentage of adipose weight relative to total body weight. * Indicates statistical significance at *p* < 0.05 correlated to the HFD group.

Following the results established in Figure 4a, the adipose weight was notably lesser in the naïve group compared to the other groups. The percentage reductions in the adipose weight were 97% for the naive group, followed by 97%, 47%, and 43% for the groups administered with Probio65 live cells, Probio-093 live cells, and *L. sakei* Probio65 SEL, respectively, when compared to the HFD group (* *p* < 0.05). There was no significant dissimilarity in adipose weight when comparing HFD-metformin, HFD-MRS, and HFD-Probio-093 SEL with the HFD group (*p* > 0.05).

Figure 4b illustrates the percent adipose fat in respect to the total body weight of the mice after the continuous consumption of HFD for 8 weeks. The HFD group exhibited an increase in the adipose fat to 94% as compared to the normal-diet-fed mice (naïve group) (* *p* < 0.05). The mice receiving Probio65 live cells exhibited a marked decreased in adipose fat of 45% as compared to the HFD group (* *p* < 0.05). The adipose fat weight in the HFD group was 5.5% of the total body fat weight. On the other hand, the mice treated with the Probio65 SEL showed only 3.2% adipose fat weight, which is 52% less than the HFD group (* *p* < 0.05). The mice supplemented with the Probio-093 SEL and live cells, metformin, and MRS exhibited no considerable difference with the HFD group.

### 2.5. Gene Expression—In Vivo

**Figure 5:**
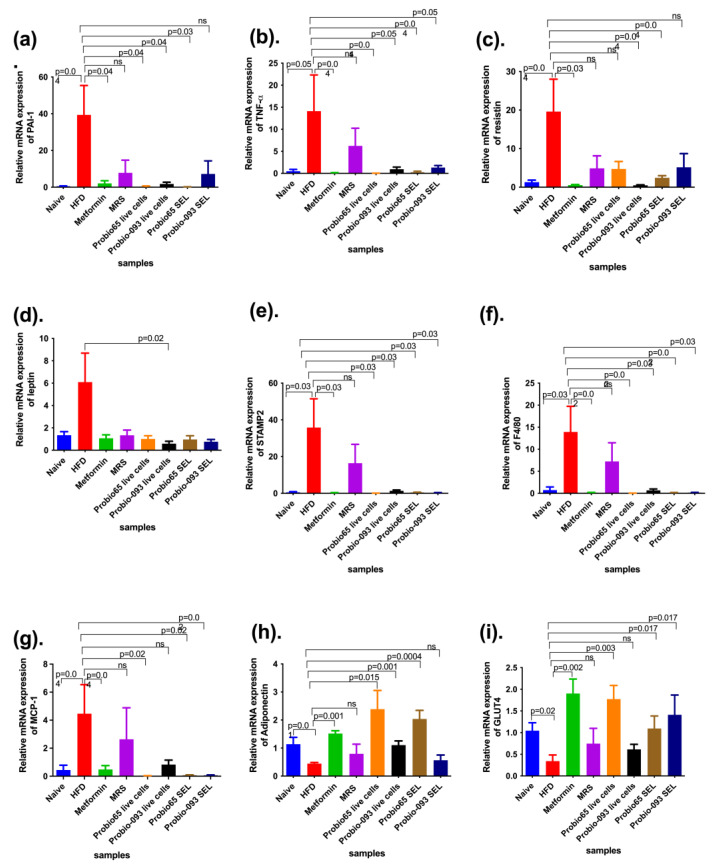
The relative expression levels of adipose tissue-related genes, including (**a**) PAI-1, (**b**) MCP-1, (**c**) TNF-α, (**d**) resistin, (**e**) leptin, (**f**) STAMP2, (**g**) F4/80, (**h**) adiponectin, and (**i**) GLUT4, were evaluated in mice undergoing 8 weeks of treatment with a high-fat diet (HFD) to induce obesity. All the gene expression results were compared to their respective controls, normalized by β-actin, and quantified as relative expression. ns indicate no sigificance.

The relative gene expression of PAI-1 in the adipose tissues of mice following 8 weeks of treatment is displayed in Figure 5a. The mice of the naïve group displayed decreased expression of PAI-1 relative to the HFD mice (*p* < 0.05). It was also observed that the mice fed with metformin, Probio65 SEL and live cells, as well as Probio-093 live cells exhibited notably lower gene expression compared with the HFD group (*p* < 0.05). The mice administered MRS and Probio-093 SEL did not show a reduction in gene expression.

The results in Figure 5b showed that the untreated mice group and mice treated metformin and the SEL and live cells of Probio65, and the SEL of Probio-093 remarkably lowered the expression of MCP-1 when compared with the HFD group (*p* < 0.05). There were no significant changes in MCP-1 expression when comparing the MRS-treated and Probio-093 live cell-treated group with the HFD group.

The relative gene expression of TNF-α over the 8 weeks of treatment is presented in Figure 5c. According to the data, the naïve mice and Probio-093 SEL- and live cell-administered mice exhibited marginally decreased TNF-α expression compared to the HFD mice (*p* = 0.05). The results also revealed that the mice provided with live cells and the SEL of Probio65 and metformin exhibited notably reduced gene expression in comparison with the HFD group (*p* < 0.05). There were no noticeable modifications in the TNF-α expression when comparing the MRS and HFD groups.

We found that the resistin expression was remarkably lower in the naïve, metformin- Probio-093 live cells- and Probio65 SEL-treated mice (*p* < 0.05) (Figure 5d). The mice receiving Probio65 live cells, Probio-093 SEL, and MRS did not display noticeable alterations as compared to the HFD group.

The relative mRNA expression of leptin in the adipose tissues of the mice after 8 weeks of treatment is displayed in Figure 5e. Among all the treatment groups, the Probio-093 live cell-treated mice exhibited a considerably lower expression of leptin when compared with the HFD group. There were no clear variations identified in any other treatment group for leptin expression in comparison with the HFD group.

Figure 5f illustrates the comparative mRNA expression levels of STAMP2 within the adipose tissues of mice subsequent to an 8-week treatment period. Notably, the control group (naïve) as well as all the treatment categories, encompassing metformin, SEL, and live cells of Probio-093 and Probio65, exhibited a consistent pattern of gene expression marked by a substantial reduction (*p* < 0.05) in contrast to the HFD group, except for the MRS group.

The relative mRNA declaration levels of F4/80 in adipose were effectively downregulated in the naïve, metformin, Probio65 live cells, Probio-093 live cells, Probio65 SEL, and Probio-093 SEL groups (*p* < 0.05) as compared to the HFD group (Figure 5g).

According to Figure 5h, the expression of adiponectin was significantly upregulated in the naïve mice and upon administration of metformin, Probio65 live cells and SEL, and Probio-093 live cells (*p* < 0.05) as compared to the HFD group. Meanwhile, the Probio-093 SEL and MRS group exhibited no significant difference as compared to the HFD group.

Figure 5 presents the proportional mRNA dissemination of GLUT4. The results indicate a significant upregulation of GLUT4 expression in the naïve group, as well as in the groups supplemented with metformin, Probio65 live cells, Probio65 SEL, and Probio-093 SEL (*p* < 0.05) compared to the HFD mice group. However, no significant irregularity in GLUT4 expression was observed between the MRS and Probio-093 live cell group and the HFD group.

### 2.6. Adipose Histology

**Figure 6:**
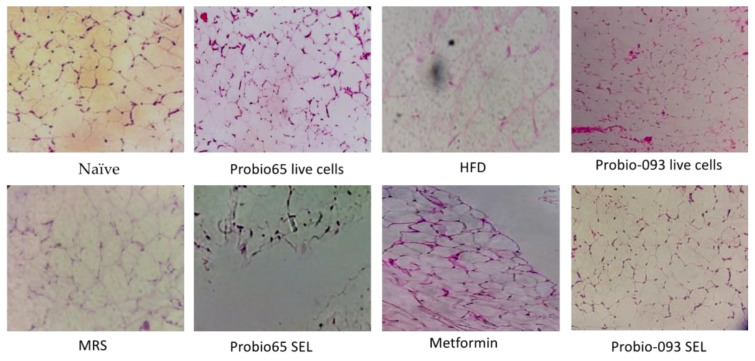
Microscopic images of mice adipose tissues stained with hematoxylin and eosin (40× magnification) were captured after 8 weeks of treatment with a high-fat diet (HFD) containing 45% kcal of fat to promote obesity. The experimental groups included naïve (a lean group of mice on a common ordinary diet, *n* = 5), HFD (an obese group of mice on a high-fat diet, *n* = 5), metformin (obese mice administered metformin, *n* = 5), MRS (obese mice administered de Man–Rogosa–Sharpe, *n* = 5), Probio65 live cells (obese mice administered Probio65, *n* = 5), Probio-093 live cells (obese mice administered Probio-093, *n* = 5), Probio65 SEL (obese mice administered Probio65 SEL, *n* = 5), and Probio-093 SEL (obese mice administered Probio-093 SEL, *n* = 5).

Figure 6 illustrates the stained adipose tissue samples from the mice. The HFD group showed significantly larger adipocyte cells, and the structural organization of the adipocyte cells was less apparent than that of the MRS group. Unlike the HFD group, the naïve group and the metformin-treated group showed only minimal cellular expansion compared to the HFD group. Intriguingly, the groups administered live cells and the SEL of Probio65 and Probio-093 demonstrated the smallest adipocytes compared to the HFD group.

## 3. Discussion

Obesity diversifies the endocrine and metabolic tissue function and results in enhanced complications that contribute to obesity such as the boosted release of proinflammatory molecules, fatty acids, and hormones [13,14,16,43]. Widespread research has indicated that fat tissues in obese individuals are a fundamental location for the formation and release of adipokines and the activation of oxidative stress signaling pathways [44]. Nutrient excess is the cause of the expansion of adipocytes which, under insulin stimulation, could actively take up glucose and store it in the form of triglycerides.

The principal objective of this study was to examine the potential anti-obesity effects of *Lactobacillus* in high-fat-diet-fed mice, coupled with in vitro analysis. The measurement of the abdominal fat mass and body weight is widely employed to assess obesity and determine the expansion of adipocytes. Our findings exhibited a significant depletion in both the body weight and adipose weight in mice following the administration of Probio65 and Probio-093. Moreover, we observed a decrease in adipocyte expansion. These results suggest that probiotics may offer promising benefits in the management of obesity.

The association between gene expression beyond lean and obese mice defines a typical reaction to the changes induced by obesity. The gene expression data show continuous variations in characteristics including adipocytes, body fat, BMI scale, and the regulation of adipose tissue function [45,46,47]. Our results indicated the decreased expression of PAI-1 by administering live cells and the SEL of Probio65 both in vitro and in vivo, and with live cells of Probio-093 in vivo and with live cells and the SEL of Probio-093 in vitro. PAI-1 itself is a glycoprotein and is known to inhibit plasminogen activation in blood and is also upregulated in obesity with an increased risk of glucose intolerance, hypertension, hyperinsulinemia, insulin hindrance, NIDDM (non-insulin-dependent diabetes mellitus), and accelerated atherosclerosis [48,49,50]. In addition, clinical trials of obese humans showed significant weight loss with surgical treatment with a control diet, and, consequently, PAI-1 expression was also reduced [49,50,51,52,53,54]. Findings suggest that some hormones or cytokines are involved in the elevated expression of PAI-1, including TNF-α, TGF-β, proinsulin, and triglycerides. Adipocytes’ central function emphasizes that TNF-α stimulates PAI-1 in mature 3T3L-1 [51,55,56,57]. In human and rodent obesity, TNF-α is elevated through the autocrine process to promote PAI-1 anabolism (biosynthesis) by adipocytes. PAI-1 mRNA expression and proteins were reduced by inhibiting TNF-α with the infusion of an inhibitor of TNF-α, pentoxifylline [58]. These data support our results showing that the expression of TNF-α was increased in the HFD-induced obesity group and reduced in the obese groups treated with the Probio65 SEL and live cells. Live cells and the SEL of Probio65 and Probio-093 also decreased the TNF-α gene expression in the 3T3-L1 adipocytes.

The expression of MCP-1 was pointedly lower in the Probio65 live cell- and SEL-administered mice and in the Probio-093 SEL-treated mice as compared to the obese mice. Such findings agreed with a previous study where adipose tissues and plasma both presented with the enhanced expression of MCP-1 in obese mice fed a high-fat diet. An increment in MCP-1 accumulation in fat tissues leads to the dispersion of macrophages in adipose tissues, insulin obstruction, and obesity-associated hepatic steatosis in mice [59]. One of the key chemoattractants for macrophages is monocyte chemoattractant protein-1 (MCP-1), from the C-C chemokine receptor 2 (CCR2) pathway, discharged by adipocytes, vascular endothelial cells, and macrophages [60]. Fat tissue macrophages release CCR2 and attract extra macrophages and monocytes and encourage the feed-forward mechanism. Inflammatory cytokines like TNF and IL (interleukin)-6 are then produced by activated macrophages.

The first adipokine discovered was leptin, in 1994, and its concentration is closely related to the whole-body fat content. Leptin is an obesity gene and expressed predominantly by adipocytes [61]. In our study, treatment with live cells of Probio-093 significantly reversed the leptin expression in contrast to the obese mice group. The in vitro SEL and live cells of Probio65 and Probio-093 changed the leptin expression compared to the NC group.

Resistin, like leptin, is emphatically connected with obesity. Resistin impacts inflammation by releasing proinflammatory cytokines [62]. Resistin promotes inflammation by inducing the development of cytokines, for example, TNF-α, IL-6, and IL-12, through a nuclear factor-Kb signal pathway in human peripheral mononuclear and macrophage cells [63]. Treatment with Probio-093 live cells and Probio65 SEL significantly reduced the expression of resistin in vivo and was lowered with live cells and the SEL of both strains in vitro. Similarly, in a previous report, a comparison was drawn between obesity and lean mice, where an increased level of resistin in plasma was found in obese mice and decreased in lean mice [64,65]. Resistin is a protein that is released from macrophages and mouse fat cells. It accelerates insulin resistance by acting on hypothalamic neurons, hepatocytes, and endothelial cells [62]. The proinflammatory effects of resistin are mediated by the pattern recognition receptor TLR4 [66].

Furthermore, we detected that, in vitro, the NC group and in vivo high-fat-diet-fed mice exhibited elevated STAMP2 and F4/80 expression, which was normalized by treatment with the SEL and live cells of both Probio65 and Probio-093. Our outcome concurs with another article’s data, and we both have demonstrated that the consumption of high-fat food is allied to the penetration of adipose tissue by macrophage infiltration (F4/80-positive cells), chemokine MCP-1 (4,41,42), and inflammatory/oxidative stress factor STAMP2 [59,67,68,69,70].

On the other hand, adiponectin is the most conspicuous anti-inflammatory adipocytokine. Our results indicated that the increased expression of adiponectin could be achieved by treatment with live cells and the SEL of Probio65 and with live cells of Probio-093 in vivo and the SEL of Probio-093 in vitro. Adiponectin and IL-10, conversely, correspond with starving plasma glucose regulation [71]. In a previous report, a high concentration of adiponectin was also correlated with the diminished occurrence of type 2 diabetes in individuals with adjustment of the body mass index, the waist–hip ratio, exercise, and smoking [72]. The concentration of plasma adiponectin is adversely linked with body fat percentage and the waist–thigh ratio [73]. Furthermore, it was discovered that adiposity linked with low circulating adiponectin is inherent with visceral expansion instead of full-body adiposity [74]. The above explanation is relevant to the fact that the overgrowth of abdominal fat has a larger impact on insulin impedance than complete obesity.

Past explorations have effectively recognized an enormous quantity of various GLUT isoforms in the liver, adipose, and skeletal muscles. GLUT4 is the main carrier for glucose transport from blood to cells and helps in energy expenditure and insulin sensitivity. Glucosamine is primarily formed over the course of hexosamine biosynthesis from glucose to fructose-6-phosphate [75]. The key passage for glucosamine into the cell may be the GLUT family of transporters. Both GLUT1 and GLUT4 have been supplied to glucosamine with equal mobility [76]. GLUT4 is the most prominent glucose conveyer to fat cells [77]. In fat cells, transgenic mice exhibiting an elevated level of GLUT4 are high-glucose tolerant and insulin-sensitive. Adipose-specific GLUT4 knockout mice exhibited basic obesity leading to entire body glucose aversion and insulin obstruction. In type 2 diabetes, GLUT4 activation in fat tissue is largely downregulated; however, it is unaltered in skeletal muscle. In our findings, GLUT4 expression was increased in the groups treated with live cells of Probio65 and SEL of both the Probio65 and Probio-093 strains as compared to the obese group of mice. Obesity and insulin resistance are linked to the reduced function of P13K in adipose tissues. For insulin-regulated glucose digestion, the phosphatidylinositol 3-kinase (PI3K)/Akt path is very important. Regulating the transport and utilization of glucose by insulin is essential for maintaining the homeostasis of whole-body glucose [78]. The binding of insulin to the insulin receptor at the extracellular domain leads to phosphorylation and a conformational change in the intracellular domain that works as a scaffold and docking site. The activation of P13K and Akt assumes a basic role in controlling and absorbing insulin-stimulated glucose by increasing the activity of GLUT4 [79]. The live cells of Probio-093 did not show effects but the SEL increased GLUT4. It is commonly recognized that probiotics have positive benefits via producing different metabolites. The metabolite of Probio93 used in this investigation is the SEL fraction, and it was concentrated before treatment. The metabolites are the primary active components of the bacteria that regulate genes; as a result, they would have a greater impact when concentrated than if the live cells were administered separately.

This study presents a holistic solution to counter the problem of obesity, with a comparable effect to conventional drugs, such as metformin. Medicinal drugs have some side effects like stomach upset, weight gain, fatigue or dizziness, diarrhea, or risk of liver diseases. Comprehensive research conducted in several nations, such as the United States, England, Brazil, and Japan, demonstrate that obesity rates are rising gradually but at extremely varied rates within each nation. In 2022, 1 in 8 people in the world were living with obesity. The health risks associated with being overweight or obese, as well as the optimal ways to manage these conditions to varying degrees, are the main foci of the recently released WHO report. This study offers a more natural way to solve this issue that is safer than drugs [80].

Probiotic-based therapies have shown promise in treating and preventing obesity, with the bulk of the evaluated research focusing on *Lactobacillus* spp. Intervention, showing significant anti-obesity effects [81]. During an eight-week period, one of the initial trials revealed that *Lactobacillus rhamnosus* PL60 decreased body weight without lowering energy consumption. Our strain-controlled obesity also lowered energy consumption by inhibiting digestive enzymes such as α-glucosidase and α-amylase, as published in our previous report [30].

Our previous [30] and current study underpins the potential of these probiotics against obesity. Nevertheless, the gut microbiota diversity and metabolic profile need to be explored in more detail, especially before and after probiotic administration. Further, studies on human populations with obesity are crucial to confirm the efficacy and safety of these probiotics for obesity management.

## 4. Materials and Methods

### 4.1. Bacterial Strain Cultivation

Two distinct lactic acid bacterial strains, designated as *L. sakei* Probio65 and *L. plantarum* Probio-093, were acquired from the microbiome laboratory at Yeungnam University, South Korea. These strains were originally isolated from the fermented Korean food, Kimchi. To maintain their viability, preserved stock cultures were stored at −20 °C in 60% glycerol. For activation, the strains were cultured in sterile MRS broth and incubated for 24 h at 37 °C [82,83,84].

### 4.2. Preparation of SEL (Ethanolic Extract)

*Lactobacillus* strains were freshly cultured and blended with an equivalent amount of 95% ethanol [85]. After 1 h of continuous shaking at 150 rpm, this culture composite was centrifuged at 4 °C for 5 min at 10,000× *g*. The vacuum was activated to remove all volatile components, resulting in a viscous pellet. Sterile phosphate-buffered saline (PBS) solution was used to dissolve the dried pellet (pH 7.4) and the resulting solution is termed ethanolic extract (SEL).

### 4.3. Cultivation of 3T3-L1 Cells into Adipocytes

Murine preadipocyte cell line 3T3-L1 was split into 6-well plates (3 × 10^3^/well) and cultivated in DMEM media containing 10% FBS (culture medium) and incubated under 5% CO_2_ environment at 37 °C [77,86,87]. In order to generate a post-confluence condition, the growth medium was subjected to vacuum twice on the first day of incubation, during the adipogenesis phase, after 48 h. The cells were then allowed to reach a state of 70–80% adipocyte condition. Then, cells were grown in adipocyte differentiation media supplemented with 0.5 mM of IBMX (3-Isobutyl-1-methylxanthine) (Sigma-Aldrich, Darmstadt, Germany), 1 µM dexamethasone (Sigma-Aldrich, Overijse, Belgium), and 10 µg/mL insulin (Sigma-Aldrich, Søborg, Denmark) for two days. On the 7th day, cells were fully in the differentiation condition for adipogenesis. After complete differentiation, the media were changed to maturation media for 2 days and at the end replaced with fresh media again (Table 1). Finally, 3T3-L1 cells were treated with SEL (100 µL/mL), live cells (10^8^ CFU) of *L. sakei* Probio65, and *L. plantarum* Probio-093 on the last day of differentiation to evaluate whether these probiotics could prevent the aggregation of intracellular triglyceride in adipocytes.

### 4.4. AdipoRed Analysis

Oil red O staining procedure was adapted for the investigation of intracellular accumulation of triglyceride (TG) [88,89,90]. The cells were cultivated in a 6-well plate under DMEM medium. Upon reaching 70% growth, the DMEM medium was changed to 2.5 mL of MDI (methyl isobutyl xanthine, dexamethasone, insulin). Furthermore, the cells were fixed with 10% formaldehyde and kept at room temperature for 10 min, followed by washing the cells twice with PBS and treating with oil red O stain. Later, the stain was washed off, and the cells were rinsed with distilled water and the extent of TG accumulation was observed under the microscope at ×20.

### 4.5. Animal Model and Diet Design

In this study, male C57BL/6J mice, aged four weeks, were used. The mice were obtained from the animal facility center of Yeungnam University, Gyeongsan, Republic of South Korea. They were housed in cages maintained at a consistent temperature and subjected to a twelve-hour dark/light cycle with a respective humidity of 60–70%. The animal experiments were conducted in compliance with approved protocols (2018-037) by the Institutional Animal Ethics Committee of Yeungnam University, adhering to appropriate procedures and standards.

A total of forty mice were randomly isolated and separated into eight groups, with each specific group consisting of five mice (*n* = 5). The mice underwent a one-week acclimatization period prior to the commencement of the experiment. All groups except the naïve group were administered a high-fat diet (45% kcal) (D12451) obtained from Opensource Diets, located in New Brunswick, NJ, USA. The objective of administering the high-fat diet was to induce obesity among the mice. The specific composition of the diet can be found in Table S1.

The ethanolic extracts (SEL) produced by *Lactobacillus* strains were derived from de Man–Rogosa–Sharpe media (MRS [the culture media used to grow the strains]); therefore, MRS was administered to one of the groups’ in vivo study to serve as a control, ensuring that if the treatment showed effects, it was the bioactive ingredients from the bacteria and not the culture media.

The eight groups were classified as follows: (1) naïve group (normal-diet-fed group), (2) HFD group (high-fat diet), (3) metformin group (HFD with metformin administered at a dose of 0.25 mg/g/day), (4) MRS group (HFD with MRS SEL administered at a dose of 5 μL/g/day), (5) Probio65 live cells group (HFD with *L. sakei* Probio65 administered at a dose of 10^8^ CFU/day), (6) Probio-093 live cells group (HFD with Probio-093 administered at a dose of 10^8^ CFU/day), (7) Probio65 SEL group (HFD with Probio65 SEL administered at a dose of 5 μL/g/day), and (8) Probio-093 SEL group (HFD with Probio-093 SEL administered at a dose of 5 μL/g/day). The administration of doses was through oral gavage. After a treatment period of eight weeks, the mice were euthanized by CO_2_ asphyxiation, and all tissue samples were carefully preserved in a nitrogen tank for other analysis.

### 4.6. Body Weight and Adipose Measurement

The changes in body weight and feed intake were observed every two weeks. The whole abdominal adipose was also measured after euthanization.

### 4.7. Gene Expression

The RNA was extracted using TRizol Reagent, according to the manufacturer’s instructions (MRC, Cincinnati, OH, USA) [30,85]. The reverse-transcribed cDNA was generated from the isolated RNA using the revertAid RTkit (Thermo Scientific, Vilnius, Lithuania). Quantification of gene expression for the targeted genes was performed using gene-specific primers (listed in Table 2) and powerSYBRgreen (Thermo Scientific, Horsham, UK) in a quantitative reverse transcriptase polymerase chain reaction (RT-PCR) system, specifically the ABI StepOne Plus instrument (Applied Biosystems, Foster City, CA, USA). All the gene expression results were compared to their respective controls, normalized by β-actin, and quantified as relative expression. The gene expression results were examined using the 2^∆∆Ct^ method. The qPCR cycle set up for initial denaturation consisted of 95 °C for 10 min, followed by 40 cycles of denaturation at 95 °C for 15 s, and annealing/extension for one minute at 60 °C. The qPCR reaction mixture included 5 μL of powerSYBRgreen, 1 μL of template DNA, and 0.5 μL of forward and reverse primers (total concentration of 10 pmol).

### 4.8. Adipose Histology

The collected samples were stored in a 10% neutral buffered formalin. The dehydration and clearing were performed manually with ethanol and xylene. In a tissue processor, the samples were embedded into paraffin overnight. Tissue samples were subsequently cut into thin sections by using a microtome. All histology sections were dyed with hematoxylin and eosin solutions and the pathophysiology was observed under a microscope at a magnification of ×40 [91].

### 4.9. Statistical Analysis

The outcomes are reported as the mean ± standard error of the mean (SEM). Statistical analysis was conducted with GraphPad Prism 10 software (GraphPad, San Diego, CA, USA). One-way ANOVA was utilized for comparing sample means within the same experiment, while an unpaired *t*-test was employed for differentiating two groups. *p*-values below 0.05 were considered statistically significant.

## 5. Conclusions

In conclusion, our study delineates the substantial anti-obesity potential of the probiotic strains Probio65 and Probio-093 with the 3T3-L1 cell line and within a high-fat-diet (HFD)-induced obesity model. Notably, the administration of the live cell fractions of these probiotics was associated with a marked reduction in body weight and adipose tissue mass in murine subjects. Additionally, the histological and biochemical assays revealed a decrease in adipocyte size and triglyceride accumulation. These beneficial effects are further corroborated by the modulation of adipocytokine gene expression, suggesting a shift towards anti-inflammatory profiles both in vitro and in vivo. Looking ahead, it is imperative to unravel the mechanistic pathways through which these probiotics exert their influence, and to investigate their long-term impact on obesity-related comorbidities including diabetes, cardiovascular diseases, and cancer. Further studies will not only deepen our understanding of probiotic-mediated pathways but also aid in optimizing strain selection and supplementation strategies. We believe the correct supplementation strategies coupled with the right environmental modifications such as in lifestyle and diet could pave the way for more effective obesity management and prevention.

## Figures and Tables

**Table 1:** 3T3-L1 cell culture and differentiation schedule.

Pre-Adipocyte Culture	Post Confluency	Adipocyte Differentiation	Adipocyte Maturation
2 days	4 days	2 days	1 day
Basic culture medium	Basic culture medium	Adipocyte differentiation medium	Adipocyte maturation medium

**Table 2:** Gene-specific primers.

Target Primers	Sequence (5-3)	Tm (°C)	References
*PAI-1*	(F) ACAGCCTTTGTCATCTCAGCC (R) CCGAACCACAAAGAGAAAGGA	59.8	[13]
*MCP-1*	(F) GCAGTTAACGCCCCACTCA (R) CCCAGCCTACTCATTGGGATCA	58.8	[13]
*TNF-α*	(F) TGGGACAGTGACCTGGACTGT (R) TTCGGAAAGCCCATTTGAGT	61.7	[13]
*RESISTIN*	(F) CTTGCCAATCGAGATGACTGT (R) GTCTGCCTGAAGCCGTGATAC	59.4	[14]
*LEPTIN*	(F) ATCTATGTGCACCTGAGGGTAGA (R) TCCTTTTCACAAAGCCACACTAT	62.9	[15]
*STAMP2*	(F) GCATCTAGTGTTCCTGACTGGA (R) TCAAATGCGGAATACCTTGCT	60.2	[13]
*F4/80*	(F) TGACAACCAGACGGCTTGTG (R) GCAGGCGAGGAAAAGATAGTGT	59.3	[13]
*ADIPONECTIN*	(F) TGTTGGAATGACAGGAGCTG (R) CGAATGGGTACATTGGGAAC	58.4	[15]
*GLUT4*	(F) CTGGGCTTGGAGTCTATGCT (R) CGCTTTAGACTCTTTCGGGC	59.3	[90]

Nucleotide symbols: R _ A or G; Y _ C or T; M _ A or C; K _ T or G; and H_ A/C/T.

## Data Availability

The data presented in this study are available on request from the corresponding author. The data are not publicly available due to patent rights.

## Supplementary Materials

The following supporting information can be downloaded at https://www.mdpi.com/article/10.3390/ph17060676/s1, Table S1: Formulation of experimental diets; rodent diet with 45 kcal% fat (D12451).
