# Supplementary material for: Efficacy of Probiotic Strains Lactobacillus sakei Probio65 and Lactobacillus plantarum Probio-093 in Management of Obesity: An In Vitro and In Vivo Analysis
Source: Pharmaceuticals (Basel). 2024 May 24;17(6):676. doi: 10.3390/ph17060676 (PMC11206994; doi:10.3390/ph17060676)
Supplement: Supplementary file 1 [file pharmaceuticals-17-00676-s001.zip › pharmaceuticals-2883479-supplementary.pdf]

Supplementary

Efficacy of Probiotic Strains Probio65 and Probio-093 in Management of Obesity: An *In Vitro* and *In Vivo* Analysis

Aneela Gulnaz[1], Lee-Ching Lew [2], Yong-Ha Park[1,2], Irfan A. Rather[3,1,*] and Yan-Yan Hor[1,2,*]

Table S1. Formulation of experimental diets Rodent Diet with 45 kcal% Fat (D12451).

Product Ingredient	g%	kcal%
Protein	24	20
Carbohydrate	41	35
Fat	24	45
Total	89	100
kcal/g	4.73	
Casein, 30 mesh	200	800
L-cystine	3	12
Corn starch	72.82	91
Maltodextrin 10	100	400
Sucrose	172.8	691
Cellulose, BW 200	50	0
Soybean oil	25	225
Lard	177.5	1598
Mineral Mix S10026	10	0
Dicalcium phosphate	13	0
Calcium carbonate	5.5	0
Potassium citrate, 1 H20	16.5	0
Potassium citrate, 1 H20	16.5	0
Vitamin Mix V10001	10	40
Choline Bitartrate	2	0
FD&C Red Dye #40	0.05	0
FD&C Blue Dye #1	0	0
FD&C Yellow Dye #5	0	0
Total	858.15	4057
